# Conserved Temporal Patterns of MicroRNA Expression in *Drosophila* Support a Developmental Hourglass Model

**DOI:** 10.1093/gbe/evu183

**Published:** 2014-09-27

**Authors:** Maria Ninova, Matthew Ronshaugen, Sam Griffiths-Jones

**Affiliations:** Faculty of Life Sciences, University of Manchester, Manchester, United Kingdom

**Keywords:** *Drosophila*, microRNAs, development, hourglass, conservation

## Abstract

The spatiotemporal control of gene expression is crucial for the successful completion of animal development. The evolutionary constraints on development are particularly strong for the mid-embryonic stage when body segments are specified, as evidenced by a high degree of morphological and protein-coding gene conservation during this period—a phenomenon known as the developmental hourglass. The discovery of microRNA-mediated gene control revealed an entirely new layer of complexity of the molecular networks that orchestrate development. However, the constraints on microRNA developmental expression and evolution, and the implications for animal evolution are less well understood. To systematically explore the conservation of microRNAs during development, we carried out a genome-wide comparative study of microRNA expression levels throughout the ontogenesis of two divergent fruit flies, *Drosophila melanogaster* and *D. virilis*. We show that orthologous microRNAs display highly similar temporal profiles regardless of their mutation rates, suggesting that the timely expression of microRNA genes can be more constrained than their sequence. Furthermore, transitions between key developmental events in the different species are accompanied by conserved shifts in microRNA expression profiles, with the mid-embryonic period between gastrulation and segmentation characterized by the highest similarity of microRNA expression. The conservation of microRNA expression therefore displays an hourglass pattern similar to that observed for protein-coding genes.

## Introduction

Numerous studies over the past decades have demonstrated that the protein-coding genes that regulate development of diverse animals are widely conserved. For example, the homeobox genes that control the anterior–posterior patterning are highly similar in their sequence, genomic organization, expression, and function in both invertebrates and vertebrates (McGinnis et al. 1984). Whole-transcriptome analyses between different species have further suggested that the temporal expression of developmental genes is well conserved (Kalinka et al. 2010; Grün et al. 2014). However, the genes expressed at different stages of embryonic development appear to be subject to different levels of constraint (reviewed in Kalinka and Tomancak 2012). A number of models have been posited to describe evolutionary constraint across development, firstly based on morphology. An early conservation model following “Baer's laws of embryology” suggested that embryos of different species are most similar at the very beginning of their development, and then gradually diverge (von Baer 1828). More recently, the “hourglass” model of embryonic conservation was proposed, where animals are most similar at an intermediate stage of embryogenesis called “the phylotypic stage,” and earlier and later stages are more divergent (Duboule 1994; Raff 1996). A number of studies have demonstrated that various aspects of transcriptome conservation follow an hourglass pattern, including gene expression variation, correlation, sequence divergence, evolutionary age, and gene regulation (Davis et al. 2005; Hazkani-Covo et al. 2005; Cruickshank and Wade 2008; Domazet-Lošo and Tautz 2010; Kalinka et al. 2010; Irie and Kuratani 2011; Quint et al. 2012; Piasecka et al. 2013). However, the early conservation model has also received recent support (Roux and Robinson-Rechavi 2008; Piasecka et al. 2013).

In the past few years, microRNAs have gained attention as alternative sequence markers for evolutionary modeling and phylogenetic analysis (Sempere et al. 2006, 2007; Wheeler et al. 2009; Rota-Stabelli et al. 2011). MicroRNAs are a class of small noncoding RNAs with well-established biogenesis and function: Mature microRNAs, generated by the cleavage of a hairpin precursor, guide the RNA-induced silencing complex to complementary sites in the 3′ untranslated regions of target mRNAs, thereby down-regulating translation (reviewed in Bartel 2004). The discovery of microRNAs revealed an entirely new layer of regulation at the level of production of proteins from mRNAs. MicroRNA regulation is known to be crucial for many processes involved in animal development: Specific microRNAs play a role in developmental timing, maternal transcript turnover, cell differentiation, morphogenesis, organogenesis, and apoptosis (reviewed in Kloosterman and Plasterk 2006). Many microRNA families are deeply conserved in bilaterian animals (Berezikov 2011), and display similar tissue specificities between divergent species, highlighting their role in the evolution of tissue identity (Christodoulou et al. 2010). For example, members of the microRNA family mir-1 are characteristic of muscle cells, while mir-9 sequences are expressed in the nervous system in both protostomes and deuterostomes (Christodoulou et al. 2010). The expression pattern of fast-evolving and lineage-specific microRNAs can also be highly conserved, as exemplified by the mir-309∼6 cluster in *Drosophila* (Ninova et al. 2014). On the other hand, detailed studies have revealed multiple instances of temporal and spatial differences in the expression patterns of some conserved microRNAs, with potential implications in animal evolution (Ason et al. 2006; Arif et al. 2013). Most studies to date concentrate on specific microRNAs, and the relationship between microRNA evolution and temporal expression conservation has not been explored in a genome-wide manner. The constraints on microRNA expression in development are therefore much less well understood than for protein-coding genes.

Deep sequencing of small RNA libraries allows the comparison of microRNA expression patterns on a global scale. We took advantage of small RNA expression data sets covering discrete time windows during the ontogenesis of *Drosophila melanogaster* and *D. virilis* to explore the temporal conservation of microRNA expression in a developmental context. These species represent two major branches in the *Drosophila* phylogeny and are at an evolutionary distance that allows assignment of orthology for both conserved and divergent microRNA genes. *D. melanogaster* and *D. virilis* are separated by approximately 63 Myr of evolution (Tamura et al. 2004), and have at least 131 microRNAs in common. We have established the timing of events during the embryogenesis of the two species, and showed that the temporal expression patterns of orthologous microRNAs are highly similar, not only for microRNAs with highly conserved sequence, but also for rapidly evolving orthologs. Globally, homologous developmental stages display well-correlated microRNA expression profiles between *D. melanogaster* and *D. virilis*. Among all periods, the stages representing gastrulation, germband elongation, and germband shortening exhibit the most similar inter-species similarity of microRNA expression, reflecting an hourglass pattern of expression conservation. This supports a model where the constraints on microRNA expression are similar to those for protein-coding genes, highlighting the integral role of microRNAs in developmental regulatory networks.

## Materials and Methods

### Embryo Collection, Immunohistochemistry, and Staging

*Drosophila melanogaster* w^1118^ and wild-type *D. virilis* stocks were maintained at 25 °C on standard media supplemented with yeast. For embryo collections, flies were allowed to lay for 2 h on apple juice agar plates with yeast paste. Adults were then removed and embryos were aged for the desired times, fixed, and immunostained using a standard protocol. We used mouse anti-engrailed primary antibody (4D9, DSHB), and Alexa Fluor 488 secondary antibody. Samples were examined using Olympus FV1000 confocal microscope, and image stacks were processed using Fiji (Schindelin et al. 2012). Homologous stages were determined by manual inspection. The morphology and the expression of the protein product of “engrailed” were assessed for a minimum of 50 embryos for each time window and representative images are presented. Embryos from each sample were found to be at a similar stage of development, ruling out gross differences due to egg retention. The immunostained 0–16 h *D. virilis* embryo samples were aliquots from the same samples used for deep sequencing (see below).

### MicroRNA Expression Data, Homology, and Sequence Divergence

We retrieved from the Gene Expression Omnibus (GEO) (Barrett et al. 2010) previously published deep sequencing libraries from our group and others, generated from samples representing different discrete developmental times in two *Drosophila* species (supplementary table S1, Supplementary Material online). These data sets include small RNA sequencing libraries from 2-h time points covering the first 16 h of *D. virilis* development, late embryos from 16 to 30 h, larvae and adult animals. For *D. melanogaster*, we retrieved multiple libraries representing four embryonic data sets (0–1, 2–6, 6–10, and 12–24 h, corresponding to the phases of cleavage divisions, gastrulation and germband elongation, germband shortening, morphogenesis, and organogenesis, respectively), larval, and mixed-sex adult data sets (Ruby et al. 2007; Chung et al. 2008).

Reads were mapped to the *D. melanogaster* microRNA sequences annotated in miRBase (v19) (Kozomara and Griffiths-Jones 2011) using Bowtie (Langmead et al. 2009) with the following parameters: -v 1 -a –best –strata. The numbers of 19–24 nt reads mapping to each microRNA, corrected for mapping to multiple loci, were used as estimates of microRNA expression levels. Eight *D. virilis* small RNA libraries representing 2-h intervals during the early (0–16 h) embryogenesis, late embryos (16–30 h), larvae and mixed-sex adults were previously generated by us using the Illumina HiSeq 2000 platform (GEO GSE54009; supplementary table S1, Supplementary Material online). Reads were mapped to the *D. virilis* microRNAs annotated by us (Ninova et al. 2014) with the same parameters as for *D. melanogaster* and counts were corrected for multiple mapping. The previously established homologous mature and hairpin microRNA sequences in the two species were aligned and sequence divergence was calculated as the number of substitutions per site in the two mature arms or the whole hairpin.

All mature microRNA read counts in all data sets were normalized as reads per million mapping to microRNAs. MicroRNAs that had fewer than ten total reads in all experiments were excluded from the analysis. Normalized read counts from separate sex adult samples were averaged to compare with mixed sex adults. Samples of *D. melanogaster* representing the same developmental time windows have closely related microRNA expression profiles, as defined by hierarchical clustering and Spearman’s correlation even when generated by different groups and on different platforms (see Results and supplementary fig. S1, Supplementary Material online), and were therefore treated as biological replicates.

MicroRNA expression similarity between different samples was assessed using the Spearman’s correlation. The 11 developmental data sets of *D. virilis* and the averaged replicates of the 6 developmental stages in *D. melanogaster* were combined in an all-versus-all manner to create all possible pairs of stages between and within species, and the Spearman’s correlation coefficient (*ρ*) was computed for each pair of stages. Error bars reflect the standard deviation from these values of the correlation coefficients obtained from comparisons with individual *D. melanogaster* replicates for each time point. Results were checked for robustness by the Euclidian distance of z-transformed RPM values as an alternative metric.

Expression time courses of orthologous microRNAs and clustered microRNAs were compared using the Pearson’s correlation (*r*) coefficient. Clusters were defined as groups of microRNAs residing on the same strand within a 10 kb distance from one another. Data were processed using custom Bash and Perl scripts, and all computations, statistical analyses, and graphs were done using R. Heatmaps and corresponding dendrograms were generated using the gplots R package.

## Result

### Timing of Events during *D. virilis* Development

**Fig. 1.—:**
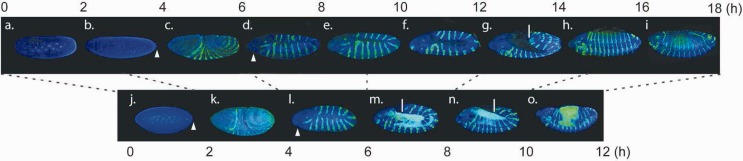
Timing of events during the first 16 h of *Drosophila virilis* development (*a–h*) compared with *D. melanogaster* (*j–o*). Samples were taken at 2-h intervals at 25 °C during the early development of the two animals until cuticle secretion starts, and stained with antibodies to detect engrailed (green). DNA is stained in blue (DAPI). Representative images for each time point are shown; embryos are oriented laterally, with anterior to the left and dorsal at the top. Dashed lines reflect matching stages assuming a 1.5-h delay in *D. virilis*. *D. virilis*: (*a*) 0*–*2 h, only the first few cleavage divisions are completed, nuclei are still localized in the middle area of the embryo; (*b*) 2–4 h, late blastoderm by the end of cleavage divisions, nuclei moved to the periphery of the blastoderm, pole bud cells are indicated by arrowhead; (*c*) 4–6 h, gastrulation and beginning of germband elongation, engrailed emerging in a periodical pattern; (*d*) 6–8 h, extended germ band, arrowhead indicates the stomodeal invagination, line shows the most posterior part of the germband, engrailed is expressed in 14 periodical stripes and in the head; (*e*) 8–10 h, extended germband; (*f*) 10–12 h, extended germband, beginning of retraction; (*g*) 12–14 h, germband retraction, line marks the posterior extent of the germband; (*h*, *i*) 14–18 h, segmented embryo, beginning of dorsal closure and head involution. *D. melanogaster*: (*j*) 0–2 h, late blastoderm, pole bud cells are indicated with arrowhead; (*k*) 2–4 h, beginning of germband elongation, engrailed is expressed in a periodical pattern; (*l*) 4–6 h, extended germband stage, arrowhead indicates the stomodeal invagination, *engrailed* is expressed in 14 stripes corresponding to each parasegment, and in the head, line indicates the germband end; (*m*) 6–8 h, head morphogenesis continues, germband retraction begins, line indicates the end of the germband; (*n*) 8–10 h, retracting germband; and (*o*) 10–12 h, segmented embryo, dorsal closure and head involution begin.

At 25 °C, *D. virilis* embryos develop for approximately 30–32 h, slower than the embryonic developmental time of 22–24 h for *D. melanogaster*. As correlating gene expression between species across development may be confounded by heterochronic shifts, we determined the timing of homologous developmental events between the two species. To this end, we immunostained aliquots of embryos from each sequenced *D. virilis* sample and from a similar *D. melanogaster* time course, and used the occurrence of key morphologically evident developmental events to identify comparable stages. Figure 1 shows representative embryos stained for nuclei and the conserved segmentation gene engrailed at each time point in the two species. In *D. virilis*, cleavage and pole bud formation occur more slowly and occupy approximately the first 4 h of development (fig. 1*a* and *b*). The first invaginations, gastrulation, and the beginning of engrailed expression occur between 4 and 6 h postfertilization (fig. 1*c*). Germband extension and associated processes are also prolonged, spanning the next 6 h (6–12 h) of development (fig. 1*d*–*g*). Germband retraction begins at the end of this period (10–12 h) and continues during the 12–14 h time point (fig. 1*h*). About 14–18 h after fertilization, the *D. virilis* embryo approaches the end of germband retraction and begins head involution and dorsal closure—a process that occurs at about 10–12 h of *D. melanogaster* development. Overall, we did not observe any significant heterochronic shifts in early *D. virilis* development, but a proportionate 1.5 times delay in the relative timing of events with respect to *D. melanogaster*. These observations are largely in agreement with the recently described notion of uniform scaling of embryogenesis in flies (Kuntz and Eisen 2014).

### Similarity of Global microRNA Expression in Developmental Intervals within and between Species

We retrieved two or more small RNA sequencing libraries covering each of the embryonic stages of 0–1, 2–6, 6–10, and 12–24 h, and larval and adult stages of *D. melanogaster*, and eight 2-h time intervals covering the first 16 h of development of *D. virilis*, 16–30 h embryos, larvae and adults (supplementary table S1, Supplementary Material online and also see Materials and Methods). These data sets contain a total of 172 and 123 microRNAs in *D. melanogaster* and *D. virilis*, respectively, each represented by 10 or more mapped reads; 118 of these microRNAs are 1-to-1 orthologs as determined by sequence similarity and synteny (Ninova et al. 2014). MicroRNAs with no homologs in the two species account for <1% of the reads at each stage. We used deep sequencing read counts as a measure of microRNA expression at each developmental stage, and compared the orthologous microRNA expression patterns between the different stages and between species using the Spearman’s correlation (*ρ*). This approach was chosen for its robustness to nonnormality, nonlinearity, and potential outliers that might have been introduced by sequence-specific biases during deep sequencing. Spearman’s correlation has often been used to assess the similarity of the protein-coding transcriptome across species (Irie and Kuratani 2011; Wang et al. 2013; Grün et al. 2014).

**Fig. 2.—:**
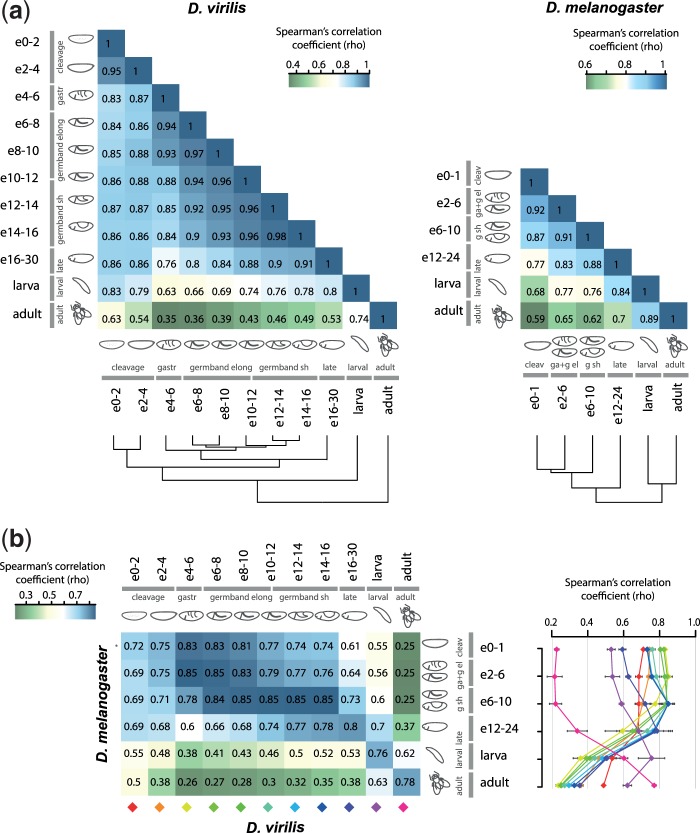
Similarity of global microRNA expression profiles between different developmental stages within (*a*) and between (*b*) the two species *D. melanogaster* and *D. virilis*. (*a*) Heatmaps representing Spearman’s correlation values for all-versus-all comparisons between different microRNA expression libraries in each of the two species: 2-h intervals within the first 16 h of *D. virilis* development (e0–2, e2–4, etc.), late *D. virilis* embryos (e16–30), larvae and adults; *D. melanogaster* 0–1, 2–6, and 6–10 h (e0–1, e2–6, e6–10), late embryos (e12–24), larvae and adults. Schematic drawings of each stage are based on morphology as determined in figure 1. Dendrograms were obtained by hierarchical clustering. (*b*) Left: Heatmap representing Spearman’s correlation values for all-versus-all microRNA expression libraries between the two species. Right: Spearman’s correlation values (*x*-axis) of cross-comparisons are plotted as colored lines corresponding to each stage of *D. virilis* (diamonds, colored as on the left panel) against each stage of *D. melanogaster* (*y*-axis); points represent average correlation values, and error bars represent the standard deviations of the coefficients for each *D. melanogaster* replicate.

We first focused on the microRNA expression profiles at different stages within each individual species in an all-versus-all manner. As expected, microRNA expression in *D. virilis* is highly correlated between neighboring stages, and the correlation gradually decreases between stages that are more distant in developmental time (fig. 2*a*, left). Adjacent time windows covering similar developmental events are better correlated than neighboring ones that represent different events in terms of morphology and function. For example, *D. virilis* samples representing early blastoderm embryos (0–2 and 2–4 h) cluster together and differ more greatly from the immediately following stages of development. We suggest that this transition represents a major shift from the presence of maternally loaded microRNAs prior to 4 h (the end of cleavage divisions), to the onset of *de novo* zygotically expressed microRNAs. The microRNA profiles in the time windows of the long germband embryo (6–8 and 8–10 h) are also closely related, and differ from the period of gastrulation (4–6 h). Differences in microRNA expression profiles are also evident among embryonic, larval, and adult stages. These results indicate that the transitions between key developmental events, maternal-to-zygotic transition, gastrulation, germband elongation, germband shortening, morphogenesis and organogenesis, larva and adult, are associated with gross changes in microRNA expression. Stage clustering of microRNA profiles therefore recapitulates the expectations from morphological observations. The *D. melanogaster* data have lower temporal resolution, but show similar trends, both for values averaged between different samples representing the same developmental interval (fig. 2*a*, right), and for individual samples (supplementary fig. S1, Supplementary Material online).

**Fig. 3.—:**
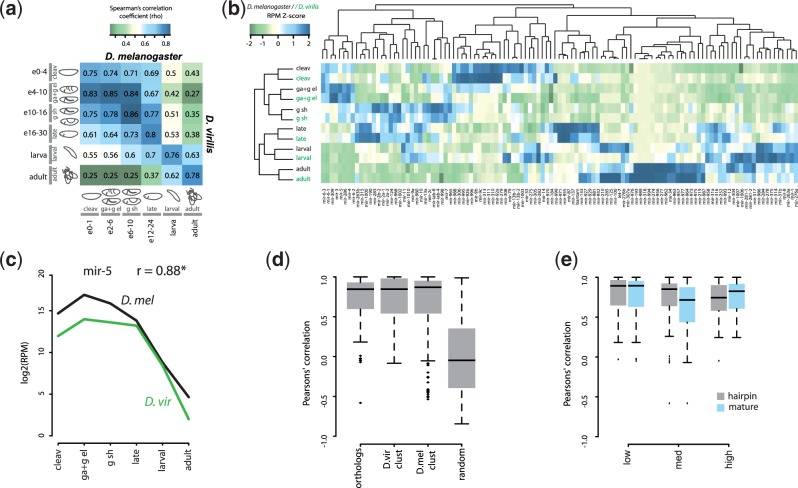
Temporal expression conservation of orthologous microRNAs between *D. melanogaster* and *D. virilis*. (*a*) Heatmap showing Spearman’s correlation values of microRNA expression for all-versus-all comparisons between pooled *D. virilis* stages (see main text) and the available *D. melanogaster* time intervals. (*b*) Heatmap showing the z-scaled expression of each orthologous microRNA at the comparable time intervals in the two species (labels are black for *D. melanogaster* and green for *D. virilis*). (*c*) Temporal expression of the mir-5 orthologs in the two species with calculated Pearson’s correlation (*r*). (*d*) Box plots representing the distributions of Pearson’s correlation of the temporal profiles of all orthologous pairs of microRNAs in the two specie*s*. *R* values for clustered microRNAs in each individual species and for randomly chosen microRNA pairs are also shown. (*e*) Pearson’s correlation of temporal profiles of orthologous pairs of microRNAs with low, medium, and high evolutionary rates in the whole hairpin and in the mature sequence.

We next addressed the similarity of orthologous microRNA expression profiles at different stages of development between the two species. Figure 2*b* shows the correlation of microRNA expression between *D. virilis* and *D. melanogaster* stages averaged between replicates (left), and the ranges of these values between individual data sets (right; heatmaps for all-versus-all data sets are presented in supplementary fig. S2, Supplementary Material online). The results show that the homologous developmental stages between *D. melanogaster* and *D. virilis* defined by key morphological events (and not time) are the most similar in terms of their orthologous microRNA expression profiles, and this is consistent between replicates. Thus, global microRNA expression across development is well conserved between species. A single exception is the very early embryo (0–1 h) of *D. melanogaster*, which is consistent between replicates (supplementary fig. S1, Supplementary Material online), but correlates slightly better with the 4–6 h embryo of *D. virilis* than the blastoderm stage. Interestingly, a similar discrepancy in the earliest stages was observed for protein-coding genes between vertebrate embryos (Irie and Kuratani 2011). Downstream analysis suggested that this is at least partly due to the presence of zygotically expressed microRNAs (e.g., from the mir-309∼6 cluster) at relatively high levels in the 0–1 h interval of *D. melanogaster*, but not in the 0–4 h interval of *D. virilis* (fig. 3*b*). The similarity of microRNA gene expression between species is also lower in the late embryo, larval, and adult stages, compared with that of the mid-embryo. Among all homologous developmental stages, microRNA expression is the most highly correlated in the intervals representing the stages of gastrulation and beginning of germband elongation (2–6 h in *D. melanogaster* data sets vs. 4–8 h in *D. virilis*), and slow germband elongation until the beginning of segmentation (around 6–10 h in *D. melanogaster* vs. 6–14 h in *D. virilis*). Therefore, the similarity of microRNA profiles across the development of *D. melanogaster* and *D. virilis* follows an hourglass pattern. The timing of the period of highest microRNA expression similarity between species starts earlier but partially overlaps with the timing of maximal protein-coding gene expression conservation in *Drosophila*, and the arthropod phylotypic stage (see Discussion). Alternative microRNA expression similarity metrics including Euclidean distance (supplementary fig. S3, Supplementary Material online), Pearson’s correlation between log2-transformed values, and values normalized by variance stabilizing transformation (Anders and Huber 2010) generate consistent results (data not shown).

### Temporal Conservation of Homologous microRNA Expression

The relatively high correlation of microRNA expression at all homologous developmental stages suggests that the temporal expression patterns of orthologous microRNAs are well conserved. To test this explicitly, we created data sets representing comparable time points by pooling the expression data from adjacent *D. virilis* developmental stages to match the granularity of available data sets from *D. melanogaster,* based on the morphological observations (fig. 1) and the within-species microRNA transcriptome similarities (fig. 2). The embryonic samples from *D. virilis* were grouped as follows: 0–4 h, cleavage; 4–10 h, gastrulation + germband elongation; and 10–16 h, germband shortening. The microRNA profiles from the resulting four embryonic data sets, together with the larval and adult, are, as expected, highly similar to the libraries covering the same stages in *D. melanogaster* (fig. 3*a* and *b*). Consistently, the microRNAs upregulated in each developmental stage are similar between the two species (fig. 3*b*). To further evaluate the degree of microRNA temporal expression conservation, we generated expression time courses for each pair of orthologs using the scaled developmental intervals, and calculated the resulting Pearson’s correlation. For comparison, we assessed the expression of microRNAs clustered in narrow genomic regions (<10 kb between adjacent genes) in each of the two species (supplementary table S2, Supplementary Material online for a full description of microRNA clusters), and randomly chosen pairs of microRNAs from the two species as a negative control. Clustered microRNAs are often coexpressed from the same primary transcript, and therefore usually have similar expression profiles (Ryazansky et al. 2011; Marco et al. 2013). Figure 3*c* shows example time courses of the mir-5 orthologs in the two species, and the distribution of the obtained correlation values for all orthologous microRNAs is shown in figure 3*d*. Overall, the temporal expression of orthologous microRNAs is highly similar between species: Mean and median values are in fact comparable with those obtained for microRNAs expected to originate from the same transcript in the same species (clustered microRNAs), and are significantly greater than those of randomly chosen microRNA pairs. This high degree of conservation holds for both intergenic microRNAs and microRNAs localized within protein-coding regions, and microRNAs localized as single genes or in clusters (supplementary fig. S4, Supplementary Material online). There is also no statistically robust correlation between microRNA temporal conservation and microRNA expression levels in each species (supplementary fig. S5, Supplementary Material online), that is, both highly and more lowly expressed microRNAs have conserved temporal expression profiles.

Orthologous microRNAs vary in their degree of sequence conservation between *D. melanogaster* and *D. virilis*; while many orthologs are perfectly conserved, some have undergone multiple substitutions in their mature sequence between the two species (Ninova et al. 2014). We tested if there is a relationship between microRNA temporal expression conservation and microRNA evolutionary rates. MicroRNAs were separated into three groups with high, medium, and low substitution rates in their hairpin sequences and the sequences of the mature arms. We find that microRNAs evolving at different rates have similar highly conserved temporal expression profiles (fig. 3*e*). There is consistently no significant correlation between microRNA evolutionary rates and microRNA temporal conservation (*r* ∈ (−0.2,0.2), *P* > 0.5). Our results therefore suggest that the temporal expression profile of a microRNA is conserved even when its sequence is not.

## Discussion

We have analyzed the temporal expression patterns of microRNAs throughout embryonic development in two Drosophilid species from two perspectives: The similarity of global microRNA expression profiles between species and the similarity of the expression profiles of pairs of orthologous microRNAs. Comparisons of microRNA expression profiles differ in many ways from comparisons of protein-coding genes and provide a unique opportunity to evaluate evolutionary trends with very different genetic elements. For example, the total number of microRNA genes in a genome is considerably smaller than genes encoding proteins, and the proportion conserved in even closely related organisms is lower: The current release of *D. melanogaster* genome (r5.54) contains 238 microRNAs, 131 of which conserved in *D. virilis*, and over 16,000 protein-coding genes, around 80% of which have annotated homologs in the current release of the *D. virilis* genome (r1.2). Unlike proteins, microRNAs are known to emerge in animal genomes by continuous acquisition throughout evolutionary time, and the proportion of lineage- and species-specific microRNAs is large (Sempere et al. 2006; Wheeler et al. 2009). Global comparisons of microRNA expression across species therefore need to be a careful compromise between evolutionary distance and availability of common data points.

Our transcriptome-wide comparison of temporal microRNA expression patterns during the development of two divergent fruit flies, *D. melanogaster* and *D. virilis*, showed that on this evolutionary scale, the temporal expression of homologous microRNAs is highly conserved. Indeed, the conservation of expression is high even when the sequences of the orthologs have diverged. This suggests that the temporal regulation of microRNA transcript expression may be more constrained than the content of the transcript itself. We speculate that this is because changes outside the seed sequence that defines target interactions have comparatively mild effects on global target regulation within a cell type. A regulatory change in an existing microRNA resulting in its misexpression in a foreign tissue, on the other hand, may result in more dramatic changes in the gene network, with deleterious consequences. This is consistent with repeated observations that ectopic expression of microRNAs outside the normal expression domain almost always results in major phenotypic consequences, while loss of microRNA function frequently has no obvious developmental consequences, even for conserved microRNAs (Bushati and Cohen 2007; Smibert and Lai 2008).

We previously reported that some fast-evolving microRNAs in *Drosophila* are expressed at high levels uniquely in the early embryo, consistent with the notion that different developmental stages are not equally robust to change (Ninova et al. 2014). A high constraint on the regulation of microRNA expression independent of sequence conservation is also compatible with this hypothesis: If some stages are more canalized or developmentally robust than others, mutations in microRNA transcripts expressed at these stages might be tolerated as long as the expression pattern is unchanged. Such mutations could include not only changes in the sequence of existing microRNAs, but also the emergence of new hairpins within the same transcript, especially when their seeds are identical to those of ancestral members. For instance, the mir-310∼313 cluster encodes a different number of mir-310 paralogs in the two species but is conserved in its maternal deposition (Ninova et al. 2014). However, changes in microRNA regulation resulting in a shift in expression to a developmental stage that is more sensitive to perturbation could be highly deleterious. The same principle can be extended to spatial expression: If some tissue types are more robust to changes in their microRNA complement, those tissues would be expected to be enriched for fast-evolving and novel microRNAs, but conserved microRNAs would maintain their tissue specificity throughout evolution. Indeed, previous studies suggested that the tissue specificity of ancient microRNAs is highly conserved (Christodoulou et al. 2010).

Given that the temporal expression of orthologous microRNAs is well conserved, it is perhaps unsurprising that homologous developmental stages in the two species also show highly correlated global microRNA expression profiles. Notably, the most highly correlated microRNA expression values are observed for samples representing the periods of gastrulation, germband elongation, and shortening, while the periods before and after these stages are less similar in their microRNA complement. Thus, mature microRNA expression profiles follow an hourglass pattern of developmental conservation. An hourglass pattern of gene expression conservation has also been observed for protein-coding genes in *Drosophila* and other organisms (Davis et al. 2005; Hazkani-Covo et al. 2005; Cruickshank and Wade 2008; Domazet-Lošo and Tautz 2010; Kalinka et al. 2010; Irie and Kuratani 2011; Quint et al. 2012; Piasecka et al. 2013), and is thought to reflect changing constraints throughout development. However, we note that the precise timing of the hourglass “bottleneck” in our comparisons overlaps with, but is not identical to, that shown at the protein-coding transcriptome level (Kalinka et al. 2010): The protein-coding gene expression conservation between species is maximal at the end of germband extension stage (8–10 h in *D. melanogaster*), while for microRNAs, the period of maximal similarity starts earlier. Furthermore, many individual protein-coding transcripts display minimal expression variation between species at the phylotypic stage (Kalinka et al. 2010). However, that pattern is not obvious for individual microRNAs, mostly because many microRNAs are present only during certain stages, and the period of minimal variance in expression often represents that of lowest abundance. Clearly, there are technical factors that obstruct the direct comparison between microRNA and whole transcriptome-based results, such as the different number of species included in the analysis, the different temporal resolution of the developmental time courses, and the different technologies employed. Nevertheless, it is likely that mRNA and microRNA expression throughout development are not subject to exactly the same constraints. For example, differences may reflect the fact that microRNAs have extremely long half-lives relative to mRNAs and proteins (median approximately 5 days compared with minutes to hours for mRNAs) (Thomsen et al. 2010; Gantier et al. 2011; Yang et al. 2003). Thus, microRNA expression may have more extended temporal effects on developmental processes. It has also been suggested that various aspects of protein-coding gene conservation do not necessarily follow the same pattern (Piasecka et al. 2013).

In conclusion, we demonstrate that key transitions during fruit fly development are accompanied by changes in the global microRNA expression profiles. The temporal dynamics of microRNA expression are highly conserved between species, particularly during the early and intermediate embryonic stages when body plans are organized. Moreover, temporal profiles of microRNAs with higher substitution rates in their hairpin and mature sequences are also similar, suggesting that the timing of microRNA expression can be more constrained than their sequences.

The conservation of transcript levels during embryogenesis provides important insights into the basic principles underlying animal development. However, as transcript abundance does not necessarily reflect protein abundance (de Sousa Abreu et al. 2009; Schwanhäusser et al. 2011), the posttranscriptional control of protein expression is an important component to consider in understanding the evolution of developmental gene expression. In a recent study, it was demonstrated that both mRNA and protein expression between two species of *Caenorhabditis* separated by approximately 30 Myr of evolution are highly correlated, while the transcript and protein levels within species are not, suggesting that gene regulation at the posttranscriptional level is highly conserved during development (Grün et al. 2014). The notion of microRNA temporal conservation during development therefore fits and complements the observed patterns of gene regulation constraints, highlighting their role as an integral part of the molecular pathways that govern animal development. On the other hand, the control of microRNA expression is dependent on transcription factors. Because of their intrinsic and complex regulatory relationships, the roles of microRNA and protein-coding gene expression are difficult to decouple. Future studies in the context of regulatory networks are therefore required to reveal a more complete picture of the mechanisms driving the development and evolution of multicellular animals.

## Supplementary Material

Supplementary figures S1–S5 and tables S1 and S2 are available at *Genome Biology and Evolution* online (http://www.gbe.oxfordjournals.org/).

Supplementary Data
