# Supplementary material for: Conserved Temporal Patterns of MicroRNA Expression in Drosophila Support a Developmental Hourglass Model
Source: Genome Biol Evol. 2014 Sep 27;6(9):2459–67. doi: 10.1093/gbe/evu183 (PMC4202322; doi:10.1093/gbe/evu183)
Supplement: Supplementary Data [file supp_6_9_2459__index.html]

Conserved temporal patterns of microRNA expression in Drosophila support a developmental hourglass model — Conserved Temporal Patterns of MicroRNA Expression in Drosophila Support a Developmental Hourglass Model — Supplementary Data 

# Conserved Temporal Patterns of MicroRNA Expression in *Drosophila* Support a Developmental Hourglass Model

## Supplementary Data

files

**Files in this Data Supplement:**

- Supplementary Data - pdf file
