# Supplementary material for: Conserved Temporal Patterns of MicroRNA Expression in Drosophila Support a Developmental Hourglass Model
Source: Genome Biol Evol. 2014 Sep 27;6(9):2459–67. doi: 10.1093/gbe/evu183 (PMC4202322; doi:10.1093/gbe/evu183)
Supplement: Supplementary Data [file supp_evu183_ninova_et_al_supplementary_data.pdf]

SUPPLEMENTARY DATA

Supplementary Figures

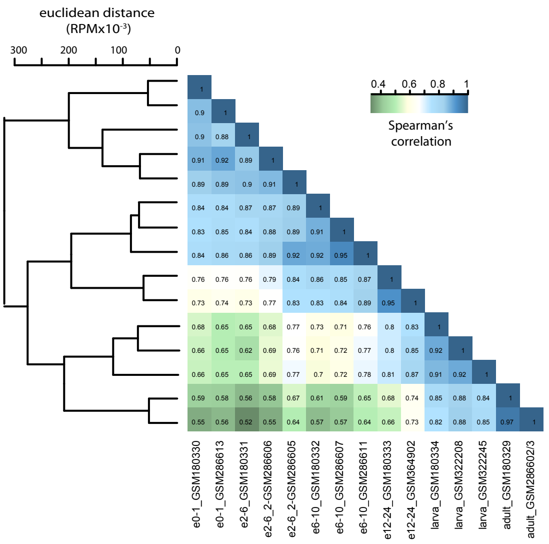

Figure S1. Heatmap showing the correlation (rho) of microRNA expression between all possible pairs of *D. melanogaster* deep sequencing datasets. Time points are labeled as in the main text, but including the corresponding raw dataset (as in Table S1). Dendrogram shows hierarchical clustering based on the Euclidean distance between samples.

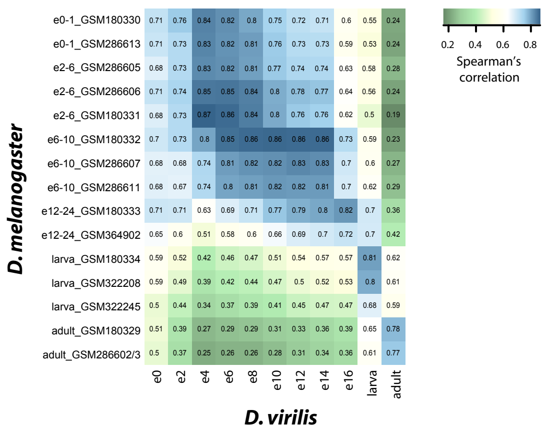

Figure S2. Heatmap representing Spearman's correlation values for all-versus-all microRNA expression libraries between the two species, where *D. melanogaster* datasets covering the same time window are considered separately.

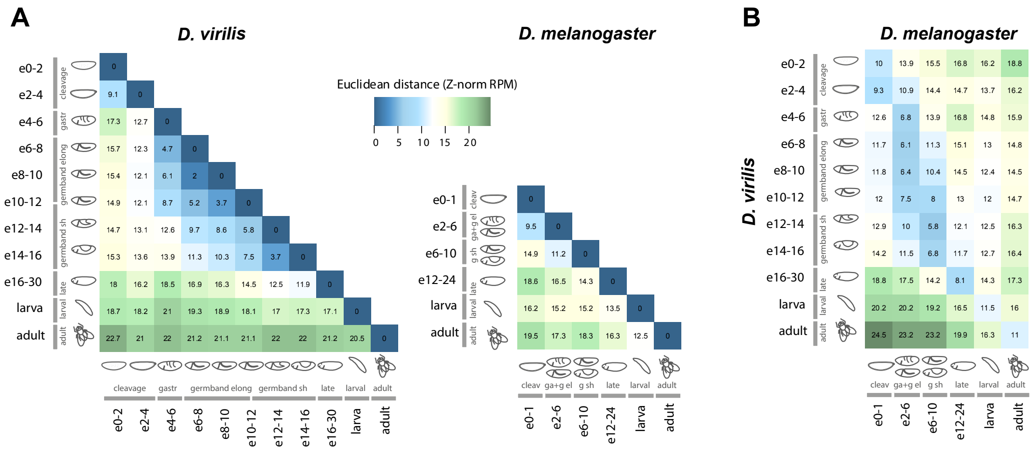

Figure S3. Heatmaps representing the Euclidean distance for all-versus-all microRNA expression microRNA expression libraries of *D. virilis* and *D. melanogaster*. A. Distance matrixes within each species: left - *D. virilis*; right – *D. melanogaster*. B. Distance matrixes between the two species. Labels are as in Figure 2 of the main text.

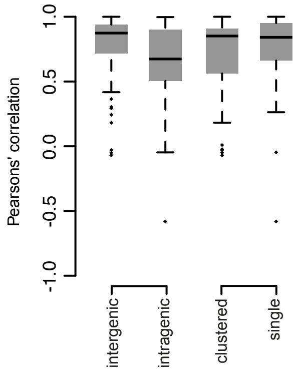

Figure S4. Distribution of Pearson's correlation values for the temporal expression of orthologous microRNAs located in intergenic regions or within protein-coding genes (intragenic) (left) and for orthologous microRNAs localized in microRNA clusters or as single entities.

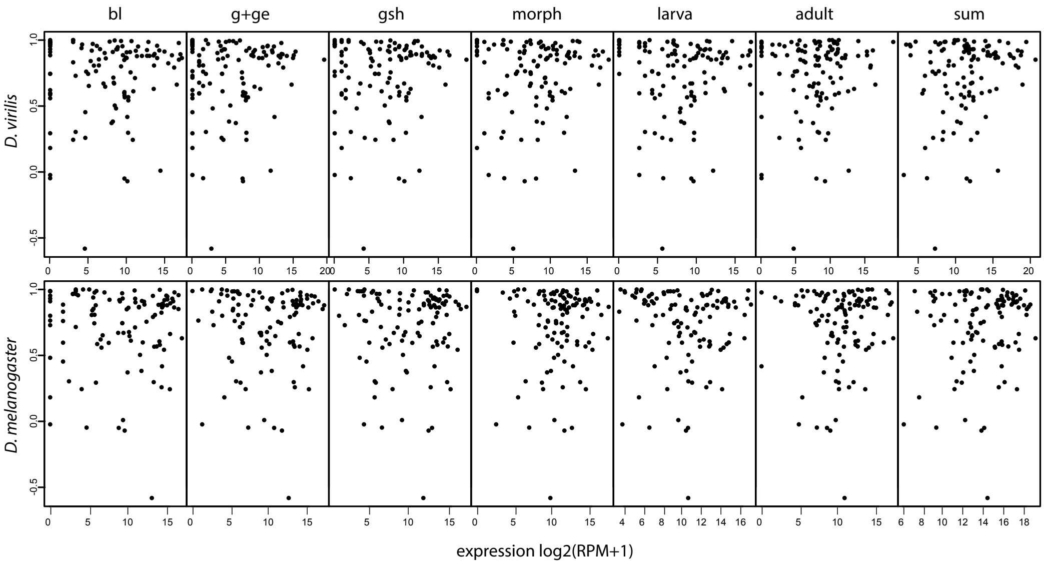

Figure S5. Orthologous microRNA temporal expression correlation values (y-axis) plotted against the expression levels of each ortholog in *D. virilis* (top) and *D. melanogaster* (bottom) at each developmental stage, or the sum expression at all stages.

Supplementary Tables

Table S1. Deep sequencing libraries used in this study.

GEO Accession	Developmental stage	Platform	Reference
GSM180330	very early embryo (0-1h)	Roche/454	(Ruby et al. 2007)
GSM286613	very early embryo (0-1h)	Illumina GA	(Chung et al. 2008)
GSM180331	early embryo (2-6h)	Roche/454	(Ruby et al. 2007)
GSM286605	early embryo (2-6h)	Illumina GA	(Chung et al. 2008)
GSM286606	early embryo (2-6h)	Illumina GA	(Chung et al. 2008)
GSM286607	mid embryo (6-10h)	Illumina GA	(Chung et al. 2008)
GSM286611	mid embryo (6-10h)	Illumina GA	(Chung et al. 2008)
GSM180332	mid embryo (6-10h)	Roche/454	(Ruby et al. 2007)
GSM180333	late embryo (12-24h)	Roche/454	(Ruby et al. 2007)
GSM364902	late embryo (12-24h)	Illumina GA	(Chung et al. 2008)
GSM322208	3rd instar larvae	Illumina GA	(Chung et al. 2008)
GSM322245	3rd instar larvae	Illumina GA	(Chung et al. 2008)
GSM180334	1st-3rd instar	Roche/454	(Ruby et al. 2007)
GSM180329	adult bodies (female & male)	Roche/454	(Ruby et al. 2007)
GSM286602	adult bodies (male)	Illumina GA	(Chung et al. 2008)
GSM286603	adult bodies (female)	Illumina GA	(Chung et al. 2008)
GSM1305819	embryo 0-2h	Illumina HiSeq 2000	(Ninova et al. 2014)
GSM1305820	embryo 2-4h	Illumina HiSeq 2000	(Ninova et al. 2014)
GSM1305821	embryo 4-6h	Illumina HiSeq 2000	(Ninova et al. 2014)
GSM1305822	embryo 6-8h	Illumina HiSeq 2000	(Ninova et al. 2014)
GSM1305823	embryo 8-10h	Illumina HiSeq 2000	(Ninova et al. 2014)
GSM1305824	embryo 10-12h	Illumina HiSeq 2000	(Ninova et al. 2014)
GSM1305825	embryo 12-14h	Illumina HiSeq 2000	(Ninova et al. 2014)
GSM1305826	embryo 14-16h	Illumina HiSeq 2000	(Ninova et al. 2014)
GSM1305829	embryo 16-30h	Illumina HiSeq 2000	(Ninova et al. 2014)
GSM1305827	3r instar larvae	Illumina HiSeq 2000	(Ninova et al. 2014)
GSM1305828	Adult bodies (male & female)	Illumina HiSeq 2000	(Ninova et al. 2014)

Table S2. MicroRNA clusters in *D.melanogaster* and *D.virilis*.

D.melanogaster	***D.virilis***
dme-mir-100, dme-let-7, dme-mir-125	dvi-mir-100, let-7, dvi-mir-125
dme-mir-11, dme-mir-998	dvi-mir-11, dvi-mir-998
dme-mir-124, dme-mir-287	dvi-mir-124, dvi-mir-287
dme-mir-133, dme-mir-288	dvi-mir-133, dvi-mir-288
dme-mir-275, dme-mir-305	dvi-mir-275, dvi-mir-305
dme-mir-279, dme-mir-996	dvi-mir-279, dvi-mir-996
dme-mir-281-1, dme-mir-281-2	dvi-mir-281-1, dvi-mir-281-2
dme-mir-283, dme-mir-304,dme-mir-12	dvi-mir-283, dvi-mir-304, dvi-mir-12
dme-mir-2b-2, dme-mir-2a-1,dme-mir-2a-2	dvi-mir-2b , dvi-mir-2a-1, dvi-mir-2a-2
dme-mir-2c, dme-mir-13a,dme-mir-13b-1	dvi-mir-2c, dvi-mir-13a, dvi-mir-13b-1
dme-mir-309, dme-mir-3, dme-mir-286, dme-mir-4, dme-mir-5, dme-mir-6-1, dme-mir-6-2, dme-mir-6-3	dvi-mir-309, dvi-mir-3, dvi-mir-286, dvi-mir-4, dvi-mir-65, dvi-mir-6-1, dvi-mir-6-2, dvi-mir-6-3
dme-mir-314, dme-mir-956	dvi-mir-314, dvi-mir-956
dme-mir-317, dme-mir-277, dme-mir-34	dvi-mir-317 /14 kb/ dvi-mir-277, dvi-mir-34‡
dme-mir-92a, dme-mir-92b	dvi-mir-92a, dvi-mir-92b
dme-mir-968, dme-mir-1002	dvi-mir-968, dvi-mir-1002
dme-mir-994, dme-mir-318	dvi-mir-994, dvi-mir-318
dme-mir-9c, dme-mir-306, dme-mir-79, dme-mir-9b	dvi-mir-9c, dvi-mir-306, dvi-mir-79, dvi-mir-9b
dme-mir-969, dme-mir-210	dvi-mir-969, dvi-mir-210
dme-mir-959, dme-mir-960, dme-mir-961, dme-mir-962, dme-mir-963, dme-mir-964	dvi-mir-962, dvi-mir-963, dvi-mir-964
dme-mir-972, dme-mir-973, dme-mir-974, mir-2499,dme-mir-4966, dme-mir-975, dme-mir-976, dme-mir-977, dme-mir-978, dme-mir-979	dvi-mir-973, candidate, dvi-mir-974-1, dvi-mir-974-2 /11kb/ dvi-mir-975, dvi-mir-976, dvi-mir-977‡
dme-mir-992, dme-mir-991, dme-mir-2498, dme-mir-313, dme-mir-312, dme-mir-311, dme-mir-310	dvi-mir-310, dvi-mir-313, dvi-mir-310hom
dme-mir-4969, dme-mir-999	-
dme-mir-984, dme-mir-983-2, dme-mir-983-1, dme-mir-303, dme-mir-982	-
dme-mir-2535b, dme-mir-1007	-
-	dvi-mir-9538, dvi-mir-9539, dvi-mir-9540
-	dvi-mir-9541, dvi-mir-9543, dvi-mir-9544, dvi-mir-9545

Homologous clusters are shown on the same line. Clusters that are completely conserved in their membership and organization are shaded in green, partially conserved clusters are in yellow, and lineage/species-specific clusters are blank.
‡ Genetic distances between microRNAs in *D.virilis* that exceed 10 kb. Despite the larger inter-microRNA distance, the organization of these clusters is clearly conserved and thus these microRNAs are considered together.
